# Bacterial Communities From Two Freshwater Aquaculture Systems in Northern Germany

**DOI:** 10.1111/1758-2229.70062

**Published:** 2024-12-15

**Authors:** Júlia Clols‐Fuentes, Julien A. Nguinkal, Patrick Unger, Bernd Kreikemeyer, Harry W. Palm

**Affiliations:** ^1^ Aquaculture and Sea‐Ranching, Faculty of Agricultural and Environmental Sciences University of Rostock Rostock Germany; ^2^ Department of Infectious Disease Epidemiology Bernhard Nocht Institute for Tropical Medicine Hamburg Germany; ^3^ Institute of Medical Microbiology Virology and Hygiene University Medicine Rostock (UMR) Rostock Germany

**Keywords:** anthropogenic activities, aquaculture, fish pathogen, freshwater, lakes, microbiome

## Abstract

The microbial communities in aquaculture systems are primarily affected by changes in water quality, fish metabolism, feeding strategies and fish disease prevention treatments. Monitoring changes in aquatic microbiomes related to aquaculture activities is necessary to improve management strategies and reduce the environmental impact of aquaculture water discharge. This study assessed the effects of activities within two fish farms on water microbiome composition by analysing the water entering and leaving both systems. Additionally, pathogenic bacterial species associated with common fish diseases were identified. The abundance, diversity and identity of microorganisms were evaluated using 16S rRNA hypervariable gene region amplicon sequencing. Proteobacteria (38.2%) and Bacteroidetes (31.3%) were the most abundant phyla in all water samples. Changes in microbiome composition after passage through the fish tanks were observed in several taxa, such as Nitrospirae, Chloroflexi, Deferribacteres and Cyanobacteria. *Flavobacterium* sp. and *Pseudomonas* sp. were the predominant potential pathogens and heterotrophic bacteria detected in both farms. Several chemolithotrophic bacteria and archaea were found in the natural reservoir used for aquaculture activities, while water microbiomes in the aquaculture systems were generally dominated by heterotrophic organisms.

## Graphical abstract

Freshwater aquaculture in open systems utilises water from a natural source, which limits the control of water conditions. Water microbiome description could indicate water quality changes and be used for improving the farm management. Water microbiome composition did not change significantly between inflow and outflow water, although heterotrophic groups tended to increase at the outflow. Discharge of aquaculture effluents may not imply negative environmental impacts. The detection of fish pathogens could anticipate fish disease events.

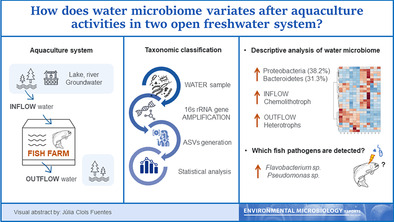

## 1 Introduction

The global use of natural freshwater reservoirs for industrial purposes such as animal production has drastically increased during the last decades leading to concerns about water quality and environmental impacts (Dodds, Perkin, and Gerken 2013; FAO 2023). Microbial communities encountered in these water bodies support ecosystem functionality since they are key participants in the nutrient cycles. The water microbial communities are usually stable and able to adapt to new conditions after a change in biological and/or environmental factors (Potts et al. 2022). Environmental perturbations can have a natural or anthropogenic origin. For instance, freshwater aquaculture often utilises the water from natural resources and returns it to the environment without being previously treated, so it could potentially perturbate the aquatic ecosystem.

Inland aquaculture represented 54.4% of the global aquaculture production, and China and Indonesia are the main producers of aquatic animals (FAO 2023). In Europe, freshwater species represent 28% of the total aquaculture production, with France, Denmark and Italy as the main producers. Germany is the country with the highest number of registered enterprises (European Commission 2023). It is one of the most relevant regions for fish species diversification, and since 2017, it had a stable inland production of new species such as sturgeon, salmonids, silver carp and african catfish. Open inland culture systems are classified as flow‐through, pond system or net cage culture depending on the infrastructure where fish are reared and the design of the installations. Flow‐through systems consist of the continuous flow of water through the enclosures where fish are raised. In this type of systems, the water is obtained from a natural source, transferred to the fish tanks, and occasionally filtered before being discharged to the natural ecosystem. The unavoidable interaction between the aquaculture activities and the ecosystem by using the source of water has several implications.

Fish farming affects the ecosystem through disease transfer or contamination of the water with disinfectants, residual antibiotics and nutrients (Naylor et al. 2000; Ottinger, Clauss, and Kuenzer 2016a). Those interactions are reflected in the water microbial community, which is influenced by changes in the water conditions (Dodds, Perkin, and Gerken 2013). The monitoring of different elements conforming open aquaculture systems, for example, the water microbiome or water quality, would give essential information for assessing the degree of impact that could cause to the natural ecosystem. Open freshwater systems exhibit singular temporal and spatial conditions. Climate and natural variability, including the seasonal variation of the water temperature, have severe implications for the productivity and management of such farms. Meteorological seasonal events, such as the rise in water temperature, are the main reason for production losses. Management operations or disinfectant treatments stresses and weakens the fish's physiology and immune system. Consequently, fish have a higher risk of suffering from parasitical, bacterial, viral and fungal infections. Infectious diseases are a major concern for the aquaculture industry, since they lead to decreased fitness and potential massive fish mortality. Fish health is also influenced by the surrounding bacterial communities in the water. A drastic change in the structure of a bacterial community may directly cause diseases in fish (Xue et al. 2017). In other cases, wounds caused by ectoparasites facilitate the coinfection with other pathogens (Okon et al. 2023). Current tools for disease control and prevention have a low effectivity and rely on chemical treatments that may disrupt the natural environment. Interpretation of the water microbiome fluctuations might open new ways to improve the efficiency of disease prevention strategies, or find new environmentally friendly ways to control pathogens.

There is the urgent need to study different aquaculture systems and to evaluate the respective microbiome community dynamics. The aim of the present study is to examine the microbial community and its variations attributed to aquaculture activities in two natural freshwater ecosystems. One system uses well water in pond systems for fish farming, and the other obtains lake surface waters for the flow through system. Presence of pathogenic bacteria in the water was also investigated and evaluated as a potential tool to improve disease preventive strategies. We exemplify two of many cases to examine possible community indicators, which could be potentially applied in other real‐world situations.

## 2 Material and Methodology

### 2.1 Source and Description of Sampling Sites

Inflow water and outflow water from two fish farms were sampled in this study, namely:

#### 2.1.1 Bimes Binnenfischerei (Frauenmark)

**FIGURE 1:**
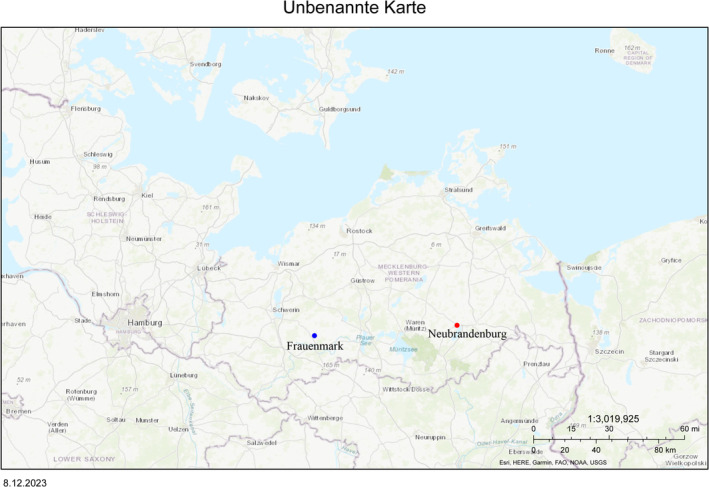
Map of northern Germany showing the state of Mecklenburg‐Western Pomerania at a central position. The two points indicate the two sites of study. The farm Teichzucht Frauenmark/Friedrichsruhe (BiMES GmbH) (53°54′47.0″N, 12°11′49.8″E) corresponds to the blue point on the map. The farm Forellenzucht Uhthoff (53°33′13.6″N, 13°14′24.8″E) corresponds to the red point.

**FIGURE 2:**
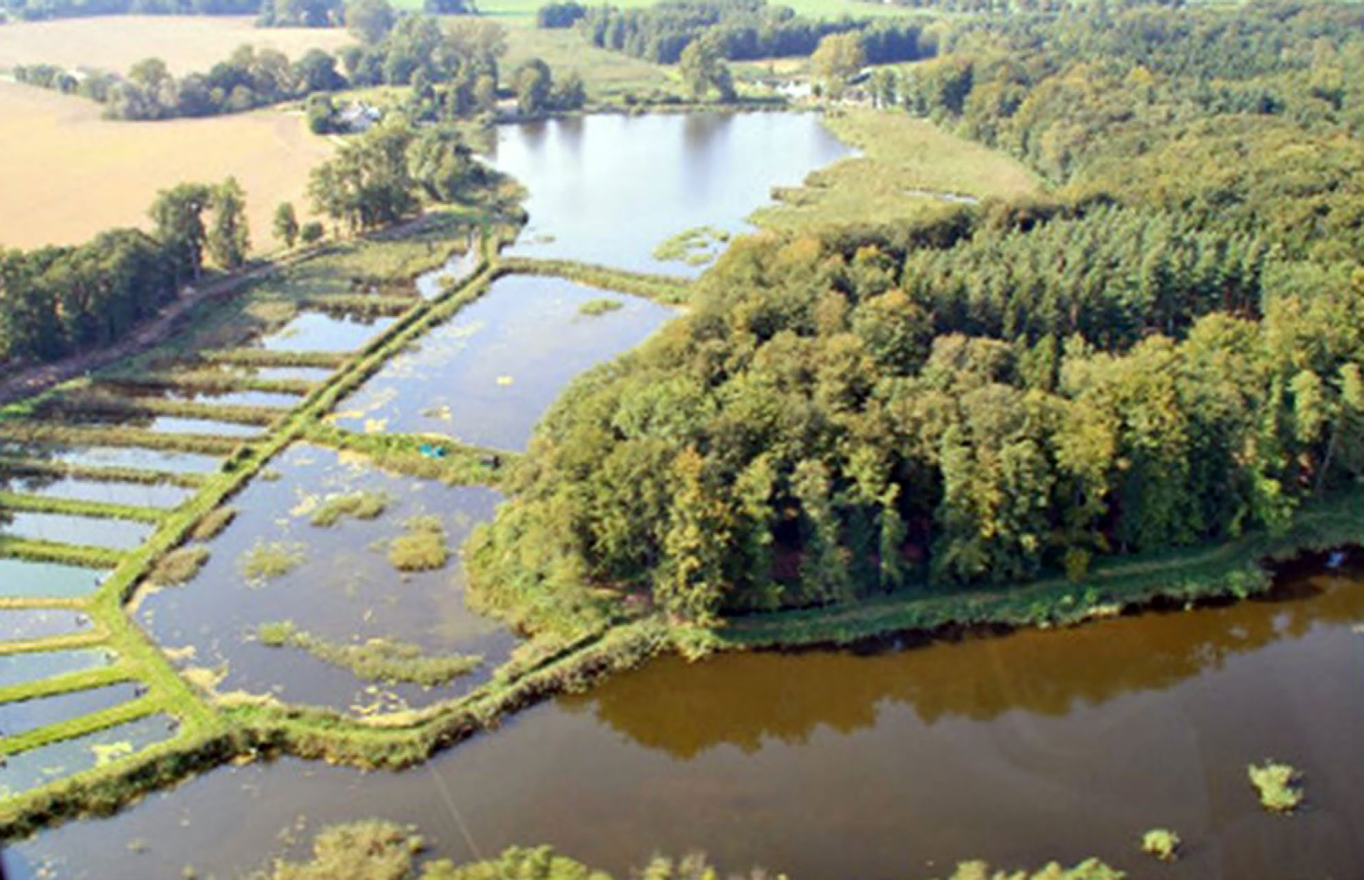
Aerial view of the pond system in BiMES, Frauenmark.

The aquaculture facility, named Teichanlage Friedrichsruhe/Frauenmark by Bimes Binnenfischerei GmbH, is located near Crivitz next to the Mühlenbach Canal, Mecklenburg‐Western Pomerania, Germany (Figure 1). The farm covers an area of about 1.5 ha, comprising more than 30 ponds, of which four are coated with concrete. Fish raised in the ponds are traditionally managed and close to the natural habitat. The facility also has long concrete raceways and a hatchery system (Figure 2). The extensive fish farming system produces adult rainbow trout ( *Oncorhynchus mykiss* ), common carp ( *Cyprinus carpio* ), whitefish ( *Coregonus maraena* ) and holds several wild fishes for restocking natural water bodies, such as northern pike ( *Esox lucius* ), european perch ( *Perca fluviatilis* ) and crayfish ( *Astacus astacus* ). Water is obtained directly from several wells in the ponds, flows through the other ponds and exits the farm by gravity. Water for the raceways is pumped from a separate well. The supply of water has a constant low temperature of approx. 10°C throughout the year. Fish are kept at low fish stocking densities. Disinfectants such as formalin and peracetic acid are commonly used for hygienic purposes and disease prevention treatment.

#### 2.1.2 Forellenzucht Uhthoff (Neubrandenburg)

**FIGURE 3:**
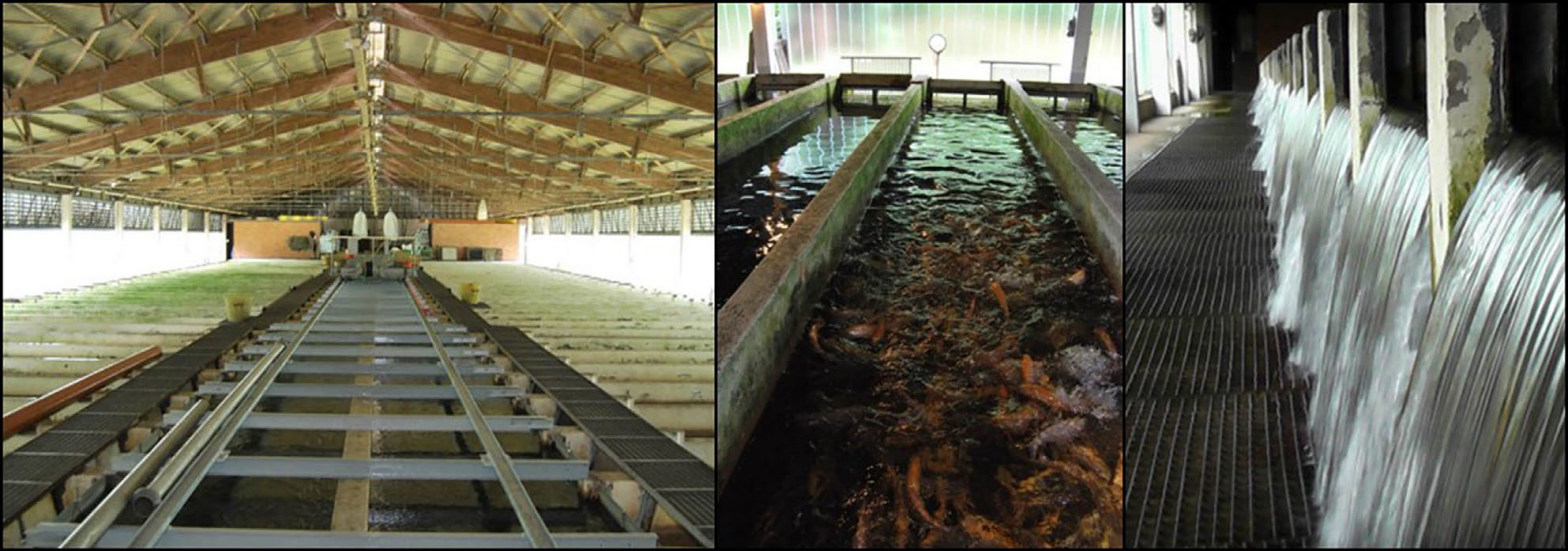
Picture of the flow through aquaculture system Forellezucht Uhthoff. The overview of the facility (on the left side), a closer view of the concrete raceways (on the centre) and an image of the water discharging from the raceway tanks (on the right side).

Forellenzucht Uhthoff GmbH is located on the northern side of Lake Tollense in Neubrandenburg, Mecklenburg‐Western Pomerania, Germany (Figure 1). Lake Tollense is an eutrophic lake with a surface area of 6550.00 ha and is considered a Site of Community Importance (SCI) by the NATURA 2000 network (Umweltbundesamt 2023). The Nature Directives for this site implies its aquatic fauna and flora conservation (European Environment Agency 2019). Surface Water is supplied directly from the Lake Tollense via the Ölmühlenbach Canal by gravity to the raceway system. Each raceway (Figure 3) has a volume of 7 m^3^ and has a constant renovation of water of several hundred L s^−1^. The fish farming system owns a hatchery facility, fibreglass tanks and raceways since the fish production covers all phases of the lifecycle. Ova are produced seasonally from a brood stock. Rainbow trout ( *Oncorhynchus mykiss* ), arctic char ( *Salvelinus alpinus* ) and sturgeon (*Acipenser* sp.) are produced in concrete raceways and distributed regionally or to their point of sale after processing the fish. The farm produces around 60–80 tons of fish per year, being summer the most productive season when they operate with high fish stocking densities. For hygienic treatments the farm commonly uses formalin 10% to disinfect equipment, empty basins and empty raceway tanks. When water temperature is above 10°C fish are weekly treated with 13%–15% peracetic acid by directly adding the disinfectant into the flow‐through raceways. The same treatment is applied during four to five consecutive days for incoming fish or fish that show any disease symptoms. As disease treatment against parasites, fish are subjected to bath treatment for 30 min with formalin 10%. The treatment against parasites is often applied to Brook trout ( *Salvelinus fontinalis* ) during autumn season.

### 2.2 Sample Collection, Storage

The systems at Neubrandenburg and Lake Tollense were sampled on the 21st of November 2018, and the system at Frauenmark was sampled on the 26th of November 2018. Triplicate water samples from representatively selected channels or ponds were collected by the aseptic collection of 1000 mL in sterile glass bottles previously sterilised in the autoclave for 20 min at 121°C. For sampling the surface water, the closed glass bottle was submerged at 30 cm depth, then the cap was opened to let the water fill the bottle. Once the bottle was completely full of water, the cap was closed and the bottle was placed in a cooler with ice. Samples for inflow water were obtained from the collection tank, before the water gets distributed to the fish tanks. Outflow water was sampled from the collection tank where water flows before getting discharged. Additionally, three samples from Lake Tollense were also sampled. Samples were filtered on the same sampling date upon arrival at the laboratory under sterile conditions using a millipore filter with the fineness of 0.22 μm (Merck Millipore KGaA, Darmstadt, Germany). The filters were stored at −20°C until further analysis.

### 2.3 Water Parameters

Temperature and oxygen concentration of the water in both aquaculture systems were measured on the sampling date with a portable multi‐meter (Hach), and the mean values for each farm were calculated (Table 1). Water parameters from Lake Tollense were obtained 2 weeks before and 2 weeks after the sampling date (Landesamt für Umwelt, Naturschutz und Geologie Mecklenburg‐Western Pomerania). The mean values for the two sampling dates were calculated (Table 1). Data for the groundwater parameters was obtained on 22 November 2018 from a monitoring station located 2.7 km away from the farm (Landesamt für Umwelt, Naturschutz und Geologie Mecklenburg‐Western Pomerania). The monitoring station collects and analyses the water at depths of 11–13 m (Point 1) and 27–29 m (Point 2) (Table 2).

**TABLE 1:** Mean values and standard deviation of the water parameters obtained at the river monitoring station No. 0331000018 (Lake Tollense) for November and December 2018.

Parameter	Lake Tollense	NB
Temperature (°C)	6.7	3.58 ± 0.25
O_2_ mg/L (%)	10.45 (84.1)	11.98 ± 1.29
pH	7.75 ± 0.07	4 ± 0.00
Total nitrogen, mg/L (%)	1.81 ± 0.21	—
Nitrite, mg/L (%)	0.03 ± 0.01	—
Nitrate, mg/L (%)	0.74 ± 0.08	—
Total phosphor, mg/L (%)	0.05 ± 0.00	—
Orthophosphate‐phosphate, mg/L (%)	0.04 ± 0.01	—
Total organic carbon (TOC), mg/L (%)	7.65 ± 0.78	—

*Note:* Mean values and standard deviation for the water parameters measured inside the aquaculture facility on the sampling date at the aquaculture system in Neubrandenburg (NB). Unknown values are represented as ‘–’.

**TABLE 2:** Values of the water parameters obtained at the groundwater monitoring station No. 24360010 and No. 24360009 (Friedrichsruhe) on November 22, 2018 and from the aquaculture farm in Frauenmark (FR).

Parameter	Point 1	Point 2	FR
Depth (m)	11–13	27–29	—
Temperature (°C)	10.2	10.1	7
O_2_ (mg/L)	5.89	0.06	6.1
pH	7.53	7.69	—
Conductivity (μS/cm)	619	738	—
Total nitrogen (mg/L)	131.787	0.495	—
Nitrite (mg/L)	< 0.03285	0.05255	—
Nitrate (mg/L)	131.786	0.443	—
Ammonia (mg/L)	< 0.064	< 0.064	—
Orthophosphate‐phosphate (mg/L)	0.076	0.047	—
Chloride (mg/L)	22	38	—
Sulphate (mg/L)	47.7	192	—
Calcium (mg/L)	97.3	127.5	—
Magnesium (mg/L)	6.28	6.44	—
Sodium (mg/L)	8.98	17.29	—
Potassium (mg/L)	1.99	1.13	—

*Note:* Unknown values are represented as ‘–’.

### 2.4 DNA Extraction, Library Preparation and Sequencing

Filters were fixed in 1 mL of Lysis buffer and DNA was extracted using the DNeasy Power Water Kit (Qiagen GmbH, Hilden, Germany) by following the manufacturer's protocol. The amplification of the V3‐V4 hypervariable region from the 16S rRNA gene was performed using the universal primers 515F (5′‐TCG‐TCG‐GCA‐GCG‐TCA‐GAT‐GTG‐TAT‐AAG‐AGA‐CAG‐GTG‐CCA‐GCM‐GCC‐GCG‐GTA‐A‐3′, including adapter sequences) and 806R (5′‐GTC‐TCG‐TGG‐GCT‐CGG‐AGA‐TGT‐GTA‐TAA‐GAG‐ACA‐GGG‐ACT‐ACH‐VGG‐GTW‐TCT‐AAT‐3′, including adapter sequences) (Klindworth et al. 2013). PCR reactions were performed for each sample and negative control with 2.5 μL of forward and 2.5 μL of reverse primers (1 μM), 25 μL of 2xKAPA HiFi HotStart ReadyMix, 15 μL of H_2_O and 2.5 μL of gDNA at 5 ng/μL. The PCR cycle was programmed with an initial 3 min step at 95°C, followed by 25 cycles of 30 s at 95°C, 30 s at 55°C and 30 s at 72°C. The last step was 5 min at 72°C. The Illumina protocol (www.illumina.com, 16S Metagenomic Library Prep Guide) was followed for the next steps, which includes purifications, indexing and clean‐up PCRs. The quality and size of the amplicon were measured using a Bioanalyzer DNA 1000 chip. Further details of the amplification protocol of the samples are described by Clols‐Fuentes et al. 2023. Illumina MiSeq sequencing produced a total of 55.745.580 reads, of which 47.881.242 reads passed filter expectations. From those, 83.5% were identified.

### 2.5 Bioinformatics and Statistical Analyses

A total of 1,595,905 raw sequencing reads were subjected to preprocessing steps, including quality trimming and filtering, in accordance with the methodology outlined in Clols‐Fuentes et al. 2023. The processing pipeline was implemented explicitly using the DADA2 workflow for amplicon sequence variant (ASV) prediction and taxonomic classification. The DADA2‐formatted 16S rRNA Silva 132 database was used to perform taxonomic assignment of ASVs from the kingdom to the genus level. A total of 1,380,722 reads were obtained after quality filtering and trimming. The sequences were deposited at the National Center for Biotechnology Information (NCBI) within the accession number PRJNA1044397.

**FIGURE 4:**
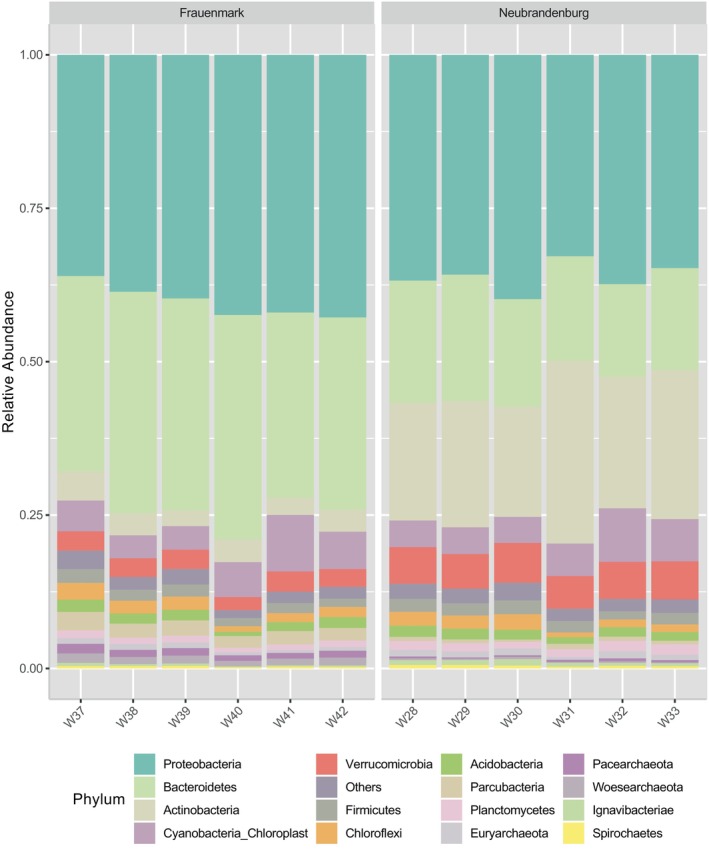
Mean relative abundance of top 15 most abundant bacterial phyla present in water inflow and outflow samples from two systems: one located in Frauenmark and another located in Neubrandenburg. Sample names appear at *x*‐axis. Samples W31, W32, W33, W40, W41 and W42 represent water outflow.

**FIGURE 5:**
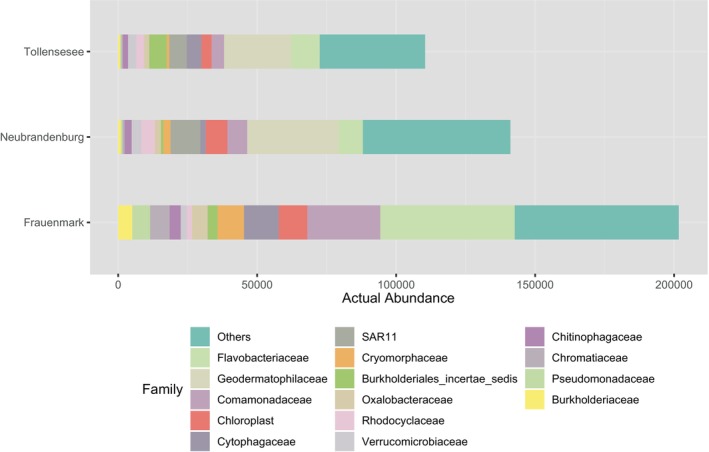
Absolute abundances of top 15 most abundant bacterial families present water samples from Lake Tollense and the aquaculture facilities located at Neubrandenburg and Frauenmark. Numbers at the bottom of the bar plot indicate the amount of ASVs.

Statistical analyses were performed at the taxonomic levels of phylum, family, and genus. Subsequent statistical analyses and visualisations were carried out with different packages in R version 4.1, following the methodology outlined by Clols‐Fuentes et al. 2023. From the total reads filtered, a total of 783,445 ASVs were identified. Based on the dominant phyla, abundance bar plots and heatmaps were generated. ASVs with significant enrichment were identified based on a false discovery rate (FDR) of less than 5% and a fold change of at least four. Significant differences were estimated using the Wilcox rank‐sum test. Abundances that were significantly different (*p* < 0.05) between sampling sites were represented on a heat tree and a bar chart showing the log_2_ fold change. Alpha and beta diversity metrics were calculated and graphically represented. A *t*‐test was performed for each site independently, where normalised counts of inflow and outflow were compared. The genera *Flavobacterium*, *Pseudomonas*, *Aeromonas*, *Mycobacterium*, *Rickettsia*, *Yersinia*, *Edwardsiella*, *Streptococcus*, *Acinetobacter*, *Arcobacter*, *Chryseobacterium*, *Shewanella*, *Lactococcus*, *Staphylococcus*, *Nocardia*, *Vibrio*, *Salmonella*, *Photobacterium*, *Renibacterium* and *Mycoplasma* were considered as potential pathogens and selected to perform abundance plots (Figures 4, 5 and 10 and Table 4).

## 3 Results

### 3.1 Water Parameters

Physicochemical parameters from the Lake Tollense showed low deviation between the two sampling points. The measurement of temperature shows lower values at both farms compared to the values obtained from the water source. The values of nitrate are very high at the near‐surface measuring point near Frauenmark compared to the other location (Table 2).

### 3.2 Ordination of Bacterial ASVs

Water bacterial community dynamics from two different aquaculture systems were evaluated and compared. On the phylum level, Proteobacteria, Bacteroidetes and Actinobacteria comprise the majority (68.7%) of the ASV abundance in all samples and both systems. The total percentage of ASVs obtained at Neubrandenburg and Frauenmark corresponded to 39.3% and 55.2%, respectively.

The 15 most abundant phyla analysed at Frauenmark belonged to the following phyla, in descending order: Proteobacteria (38.2%), Bacteroidetes (31.3%), Cyanobacteria (5.1%), Actinobacteria (3.9%), Firmicutes (3.8%), Verrucomicrobia (3.0%), Acidobacteria (2.5%), Parcubacteria (2.3%), Chloroflexi (1.9%), Planctomycetes (1.4%), Woesearchaeota (1.3%), Pacearchaeota (1.2%), Euryarchaeota (0.6%), Ignavibacteriae (0.3%) and Spirochaetes (0.3%) (Figure 4). The following distribution of ASVs were obtained at Neubrandenburg, in descending order: Proteobacteria (40.2%), Bacteroidetes (18.0%), Firmicutes (7.5%), Actinobacteria (4.1%), Verrucomicrobia (4.6%), Cyanobacteria (3.5%), Chloroflexi (2.4%), Acidobacteria (2.5%), Parcubacteria (2.3%), Planctomycetes (2.6%), Woesearchaeota (1.6%), Pacearchaeota (1.0%), Euryarchaeota (0.9%), Spirochaetes (0.3%) and Ignavibacteriae (0.3%) (Table 3 and Figure 4). The 15 most abundant families were represented to obtain a visual comparison between the natural water system and the aquaculture systems (Figure 5). The profile of absolute abundances on the family level from Lake Tollense showed a similar pattern than the samples obtained at Neubrandenburg, being Flavobacteriaceae, Geodermatophilaceae, SAR11 and Comamonadaceae the predominant families of the community (Figure 5).

**TABLE 3:** Phyla distribution in the two locations.

	Neubrandenburg (%)	Frauenmark (%)
Proteobacteria	40.18	38.24
Bacteroidetes	18.21	31.27
Actinobacteria	4.08	3.87
Cyanobacteria	3.54	5.15
Verrucomicrobia	4.61	2.98
Firmicutes	7.50	3.82
Chloroflexi	2.43	1.87
Acidobacteria	2.50	1.46
Parcubacteria	2.31	2.26
Planctomycetes	2.61	1.43
Pacearchaeota	1.04	1.25
Woesearchaeota	1.65	1.29
Chlamydiae	2.23	0.24

*Note:* Data indicate relative abundances (percentages of ASV counts). Phyla with relative abundances lower than 1% were filtered out.

### 3.3 Bacterial Diversity and Community Composition

**FIGURE 6:**
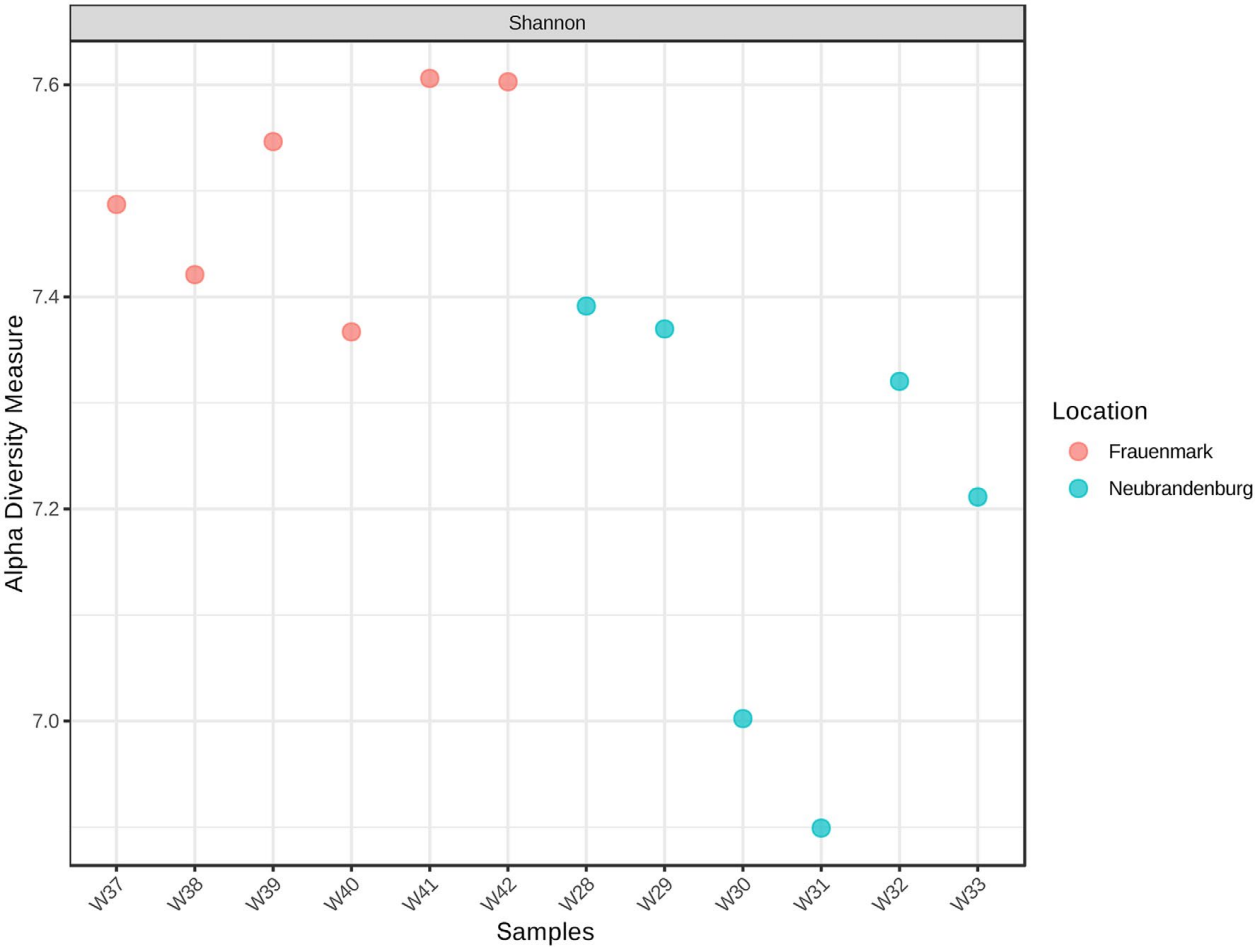
Plot summarising Shannon Alpha‐Diversity indexes obtained at each sample point and site; Frauenmark samples are marked in red colour, and Neubrandenburg samples are marked in blue. Sample numbers W28, W29, W30, W37, W38 and W39 represent water inflow. Samples W31, W32, W33, W40, W41 and W42 represent water outflow.

Alpha diversity values were calculated separately for each sampling site to provide with community descriptives. Values of Shannon alpha‐diversity tended to be higher at sample points obtained in Frauenmark, ranging from 7.35 to 7.6, compared to Neubrandenburg where values range from 6.9 to 7.39 (Figure 6 and Figure S1). The Beta‐Diversity Index differed between location sites, which resulted in two different clusters, one with the samples from Neubrandenburg and the other with samples from Frauenmark (Figure S2).

**FIGURE 7:**
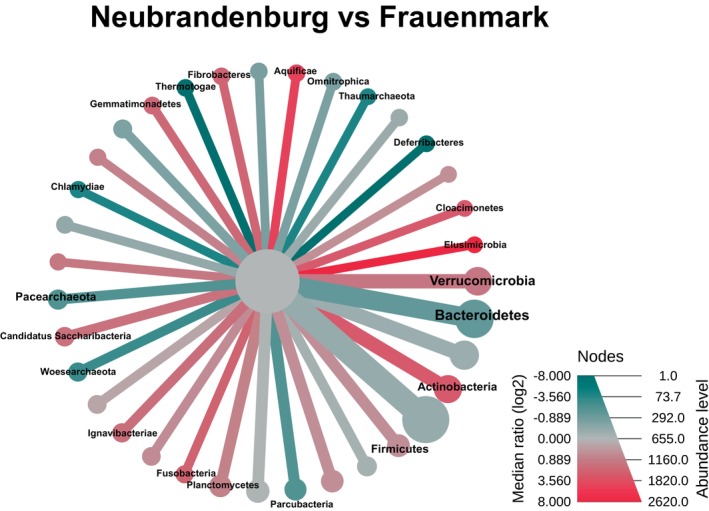
Heat tree shows pairwise comparisons of the two water microbiome communities, one sampled at Neubrandenburg and one at Frauenmark. The plot only shows the significant differences under the cut‐offs log_2_ (NB/FM) > 2 and *p* < 0.05. The colour of each phylum represents the log_2_ ratio of median proportions of the reads obtained at each site. Red colour denotes for high abundance values in Neubrandenburg compared to Frauenmark. Green colour represents high abundance values in Frauenmark compared to Neubrandenburg. Node diameter and branch width represents the number of ASVs classified as the taxon mentioned.

**FIGURE 8:**
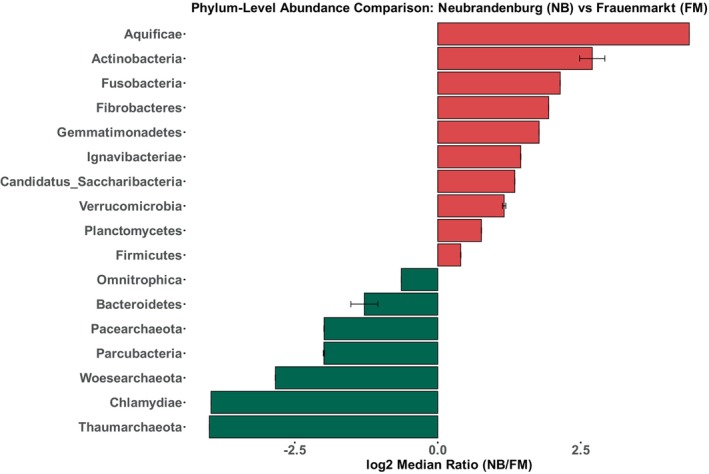
Bar chart showing mean log_2_ (fold change) of the phyla that are significantly more abundant in Neubrandenburg (in red) and in Frauenmark (in green). The plot only shows the significant differences under the cut‐offs log_2_ (NB/FM) > 2 and *p* < 0.05. The significantly abundant phyla from Neubrandenburg and Frauenmark are represented in red and green colour respectively.

A heat tree was obtained from the comparison of the two aquaculture systems to visualise the statistically different abundances between sites (*p* < 0.05) (Figure 7). For example, Thermotogae, Deferribacteres, Chlamydiae and Woesearchaeota were enriched in Frauenmark (in green) in comparison to Neubrandenburg, where the abundance of Aquificae, Elusimicrobia and Actinobacteria was higher (in red). The bar chart shows the difference between locations of those taxa that had the lowest significance level (*p* < 0.05) (Figure 8). For instance, the abundance of Thaumarchaeota was significantly higher in Frauenmark compared to Neubrandenburg.

**FIGURE 9:**
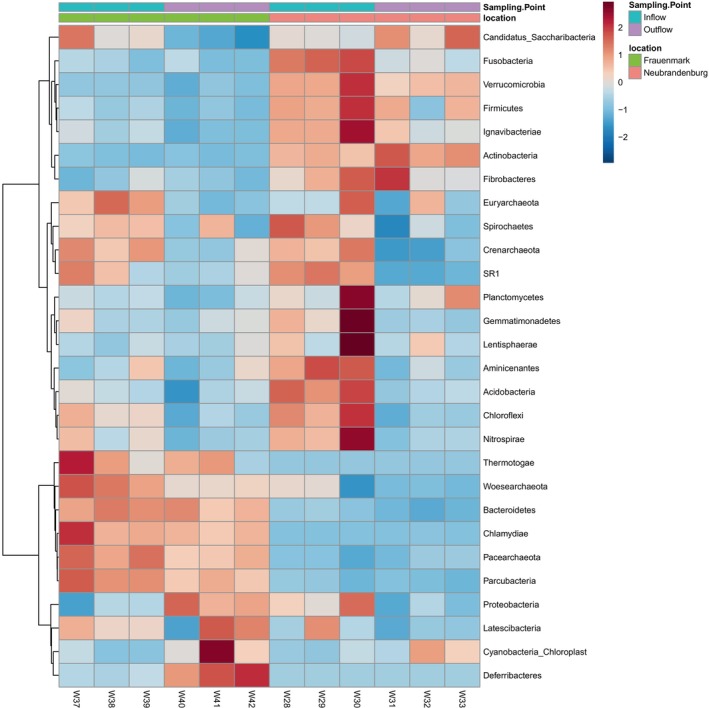
Heatmap showing the relative abundances of the phyla from water inflow and outflow at Frauenmark and Neubrandenburg systems. The taxonomy appears on the right. Differential abundances appear in two colours, blue and red, corresponding to low and high abundance of ASVs in the phyla respectively. High intensity colours denote in which sample the phyla were more abundant. The legend on the right indicates the range of colours that represent each abundance (from −2 to 2) and the types of systems and samples. Frauenmark samples are the six columns from the left, and Neubrandenburg samples are the other six columns on the right side. Sample numbers W28, W29, W30, W37, W38 and W39 represent water inflow. Samples W31, W32, W33, W40, W41 and W42 represent water outflow.

A heatmap was created to gain further insights into changes within each aquaculture system related to inflow and outflow water. Results from the *t*‐test between inflow and outflow showed no significant differences (*p* > 0.05). Based on the heatmap (Figure 9), it can be observed in more detail how the bacterial community composition differed in both systems. The heatmap lists phyla that have the biggest differences in abundance between in‐ and outflow water independently of their level of dominance within the microbial community. In Frauenmark's aquaculture system, the phyla that appeared to be of greater abundance were Cyanobacteria in the outflow water, and Thermotogae, Chlamydiae and Woesearchaeota in the inflow water. Most phyla showed a decrease in abundance from the inflow to the outflow water, except Cyanobacteria, Deferribacteres, Latescibacteria and Proteobacteria, whose abundances increased in the outflow water. The same decreasing trend regarding several phyla was observed in Neubrandenburg, with other exceptions such as Candidatus Saccharibacteria, Actinobacteria or Cyanobacteria. Up to eight phyla seemed to have an increased number of ASVs in the inflow compared with the outflow water. The abundance from several taxonomic groups was highly variable between triplicates.

### 3.4 Pathogenic Bacteria

**FIGURE 10:**
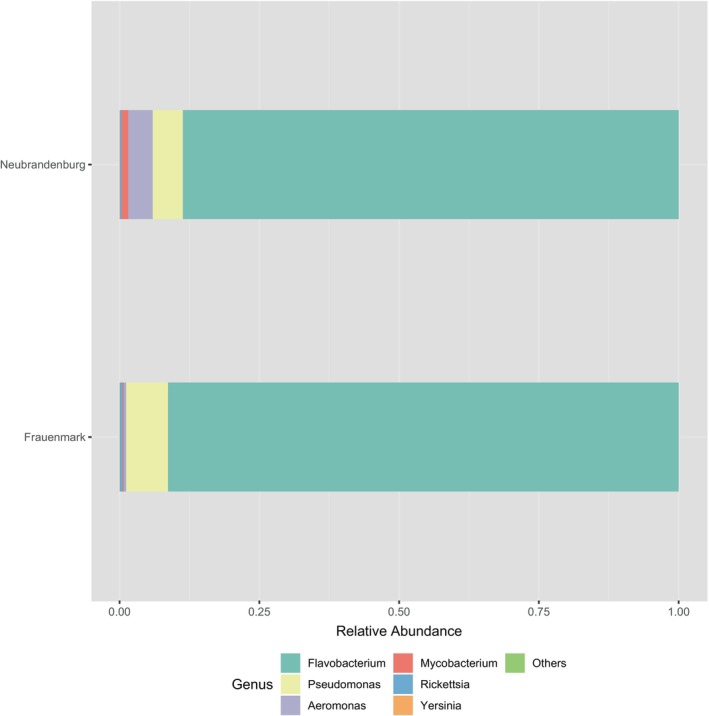
Relative abundances corresponding to several bacterial genera known as potential fish pathogenic genera. Values belong to all sample points from water inflow and water outflow at the sites Frauenmark and Neubrandenburg. Each colour denotes for a pathogenic bacterial genus.

In order to detect potential fish pathogens in open‐water freshwater aquaculture, genera of the most commonly reported bacterial species that are causative agents of fish diseases were separately analysed for Frauenmark and Neubrandenburg (Table 4 and Figure 10). The abundance of ASVs that corresponds to pathogenic bacteria reached the highest values in samples from Frauenmark, with the genus *Flavobacterium* (average 15%) as the main contributor, followed by *Pseudomonas* (1.3%) (Table 4). *Flavobacterium* had also a higher contribution than other genera in most sample points from Neubrandenburg (Table 4). Other potentially pathogenic genera, which are *Pseudomonas* sp., *Aeromonas* sp., *Mycobacterium* sp., *Rickettsia* sp., *Yersinia* sp., *Edwardsiella* sp. and *Streptococcus* sp., corresponded to 1.17% of the total ASVs counts in Neubrandenburg.

**TABLE 4:** Genera distribution of fish pathogens in the two locations.

	Neubrandenburg	Frauenmark
*Flavobacterium*	2.22%	14.93%
*Pseudomonas*	0.52%	1.25%
*Aeromonas*	0.10%	0.07%
*Mycobacterium*	0.15%	0.17%
*Rickettsia*	0.14%	0.09%
*Yersinia*	0.06%	0.02%
*Edwardsiella*	0.15%	0.04%
*Streptococcus*	0.04%	0.00%
*Acinetobacter*	0.26%	0.25%
*Nocardioides*	0.04%	0.02%
*Arcobacter*	0.20%	0.86%
*Chryseobacterium*	0.10%	0.05%
*Lactococcus*	0.02%	0.00%
*Shewanella*	0.11%	0.15%

*Note:* Data indicate relative abundance (percentages of ASV counts). The genera with less than 0.1% of ASV counts were filtered out.

## 4 Discussion

Bacterial communities from natural freshwater sources constitute a large reservoir of genetic diversity, which should be valued when aquaculture practices depend on and interact with natural water sources. Water is the link to both natural and artificial closed systems, and its physicochemical and biological parameters may be influenced by anthropogenic activities and natural phenomena (Obieze et al. 2022). Two freshwater systems were studied, one located in Frauenmark and another located in Neubrandenburg. All in all, the water community was dominated by Proteobacteria, Bacteroidetes, Actinobacteria and Cyanobacteria (Table 3), similarly to other freshwater bodies previously studied (Li et al. 2023; Obieze et al. 2022; Wei et al. 2023; Zemskaya et al. 2020). Water microbiome communities of those farms were spatially separated and defined by different biochemical, physical and biological processes. Thus, both bacterial communities differed in quantitative, qualitative and diversity parameters. For instance, beta diversity measures evidenced two single clusters, suggesting that there is a high inter‐variability between locations (Figure S2). Additionally, certain phyla statistically differed (*p* < 0.05) between farms, such as Aquificae, Actinobacteria, Chlamydiae and Woesearchaeota (Figures 7 and 8). SAR 11 was only detected in Neubrandenburg and Lake Tollense (Figure 5). SAR11 is the most abundant bacterioplankton in the oceans and freshwater bodies (Henson et al. 2018), so it is a clear example of how the distribution of some bacterial taxa are restricted to a certain habitat (Yachi and Loreau 1999). The microbial communities from Lake Tollense and the aquaculture farm in Neubrandenburg showed similar proportions of abundance in most of the taxa (Figure 5), which is plausible since the water used at the fish farm is obtained from that lake. Diversity values are descriptors of the whole bacterial community and provide information about the structure of the community. Similar diversity values at the two studied farms (Figure 6 and Figure S1) could indicate that the microbial communities were slightly dominated by a few phyla, and numerous groups that had very low abundance values (Yachi and Loreau 1999). Communities with high biodiversity are more resilient to environmental changes, since a greater number of species guarantees that some will still maintain a certain function even if other species disappear (Yachi and Loreau 1999). The effects of freshwater aquaculture systems to the natural environment are difficult to stablish and depend on several factors, such as the intensity of the fish production, climatological events or the farming management. In some cases, the impacts attributed the farming activities is very low and seasonal variations are the main force defining the water microbiome dynamics (Marmen et al. 2021), whereas other studies reported a negative impact to the water microbiome of the natural ecosystem (Zhang et al. 2020; Ottinger, Clauss, and Kuenzer 2016b). Modulations in the composition of the community between sampling points (inflow, outflow) were observed at lower taxonomical levels (Figure 9) in both farms, but not statistically significant (*p* > 0.05), which might indicate the aquaculture activities have a very low impact on the functionality of the water microbiome. It is of great complexity to stablish the causes of the microbial community shifts in diverse water bodies, and to find indicators that might be useful for the improvement of aquaculture practices. Microbiome modulations of each sampling site are discussed separately in the following sections.

### 4.1 The Study Site in Frauenmark

The farm located at Frauenmark uses groundwater for current aquaculture practices throughout the year. Near‐surface aquifers in Mecklenburg‐Western Pomerania often lack of dissolved oxygen compared to deeper groundwater, as it was measured at Point 1 (Table 2) (Kadnikov et al. 2020), which results in the shift of nutrient availability when the water migrated to the surface (Table 2). For instance, the presence of nitrate in the water was higher than 100 mgL^−1^ at deeper oxidising conditions (Point 1, Table 2), whereas nitrate was not measured at near‐surface water (Point 2, Table 2). These values are commonly measured at the studied region, and were reported in previous years (Bundesministerium für Umwelt, Naturchutz, nukleare Sicherheit und Verbraucherschutz 2020). The area of Frauenmark has a prominent agricultural activity and the use of nitrogen fertilisers in the field is commonly a source of nitrates which pollute the groundwater (Bijay‐Singh and Craswell 2021). The biochemical processes that take place at the soil and groundwater can lead to the reduction of nitrate when oxygen is not present (Cremer 2015). At the farm of Frauenmark the water is pumped from the well and collected to different ponds before it flows by gravity to the fish tanks. Thorough this process, water is oxygenated at the surface and becomes less suitable for anaerobic microorganisms. For instance, phyla such as Woesearchaeota (Liu, Wang, and Gu 2021), Nitrospirae (Mehrani et al. 2020), Crenarchaeota (Dawson, Delong, and PACE 2006) or Parcubacteria (León‐Zayas et al. 2017), that anaerobically consume carbon, nitrogen and/or sulphur, were highly abundant at the inflow water but tended to decrease in the outflow (Figure 9). Exceptionally, the strict anaerobic members of Deferribacteres increased at the outflow (Figure 9). *Caldithrix* sp. was the most abundant genus identified within Deferribacteres. This genus was identified in diverse low‐oxygen habitats, such as aquatic sediments (Yu et al. 2016) or wastewater (Chen et al. 2021; Yin et al. 2019), and are capable to reduce nitrate. Deferribacteraceae members are also known to be capable of anaerobically degrading persistent organic pollutant compounds and this family has been proposed as an indicator for the presence of oil in fish gut microbiomes (Walter, Bagi, and Pampanin 2019). The common use of machines for farming activities and material transportation could be a potential source of organic pollutants. Another factor to discuss could be that the fish faeces and uneaten feeding pellets sink and accumulate on the pond floor (Coldebella et al. 2018; Boyd 2011), resulting in the creation of anaerobic micro‐environments rich in nutrients (Santander‐de Leon et al. 2017) and the detection of Deferribacteres at the water.

Other microorganisms that are capable of degrading aerobically organic and inorganic substances produced at the aquaculture system would increase in abundance. Some of the groups that better reflect this change between the inflow and outflow water and are the phyla Proteobacteria, Latescibacteria (Farag, Youssef, and Elshahed 2017) and Cyanobacteria. Cyanobacteria was the third mostly abundant phylum in samples from Frauenmark (Figure 4), and appears to be a dominant phylum in other studies from natural inland waters (Zemskaya et al. 2020). When using SILVA database for the microbiome analysis, chloroplasts are classified inside the Cyanobacteria phylum due to the evolution of this organelle (Raven et al. 2003). These photosynthetic organelles are also present in other eukaryotic cells, such as microalgae and algae. Some microalgae can pass through the pore of size 0,8 μm (Ahmad et al. 2013). Therefore, the abundance of cyanobacteria represents then the abundance of chloroplast, which includes cyanobacteria and eukaryotic organisms. A rise in atmospheric CO_2_ and nutrient concentrations (in the form of nitrate and phosphate) benefit the growth of cyanobacteria and other photosynthetic organisms (Brookfield et al. 2021; Ma et al. 2019). A water enrichment in dissolved metabolites such as ammonia, urea, and organic matter, is commonly reported at aquaculture facilities (Ottinger, Clauss, and Kuenzer 2016b). High number of ASVs corresponding to Cyanobacteria were specifically detected at the outflow water compared to the inflow water (Figure 9), indicating that the conditions inside both aquaculture systems were beneficial for the proliferation of algae. Algal blooms can cause major problems for the water quality, such as turbidity increase, hypoxia and anoxia of water, death of the living fish and benthic invertebrates, and animal intoxication due to the cyanotoxins that some genera produce (Amorim and Moura 2021; Hallegraeff, Enevoldsen, and Zingone 2021; Huisman et al. 2018). Therefore, the maintenance of adequate water conditions is important for preventing algal blooms and further problems associated with these events. Additionally, Cyanobacteria serve as feed for some protozoan parasites like the genus *Trichodina* and have previously been connected with water eutrophication (Palm and Dobberstein 1999a). They proliferate and cause major infections on fish and other aquatic animals (Lom and Dyková 1992; Madsen, Buchmann, and Mellergaard 2000). Parasitological studies detected *Trichodina reticulata* in fish from Frauenmark, where a study on the parasites' fauna was conducted in 2018. Consequently, the high abundance of Cyanobacteria could support the growth of trichodinid parasites with further negative consequences on fish health and welfare (Barber 2007; Palm and Dobberstein 1999b).

### 4.2 The Study Site in Neubrandenburg

The water used at the aquaculture facility in Neubrandenburg is obtained directly from Lake Tollense Lake surface water, because it reaches the farm through an inflow canal. Therefore, we observed a comparable microbiome profile regarding the predominant bacterial phyla in the farm and the lake (Figure 5). Lake Tollense is described as a eutrophic lake. The nutrient levels of that type of lake change seasonally and depend on fluctuations in the thermally stratified layer (Sharma et al. 2019). Nutrients accumulate at the bottom, so usually the sediments are rich in organic matter. The upper layer of the water is warmer and oxic during summer, while the bottom layer is colder and usually deficient in oxygen. There is mixing between these strati during the autumn when temperature decreases, and then community structure might change (Sharma et al. 2019). As a result, the upper layers are enriched by the nutrients from the sediments. These events can give an explanation for the high abundance of some bacterial groups in the inflow water that typically colonise anoxic habitats and sludge, such as Crenarchaeota or Firmicutes (Kadnikov et al. 2020; Mhete et al. 2020) (Figure 9). The dominant type of Crenarchaeota consisted of microorganisms of the orders Desulfurococcales and Thermoproteales, which have been previously isolated from hyperthermophile habitats, mudholes or acidic soils (Dworkin et al. 2006; Yrjälä and Lopez‐Echartea 2021), and also in very low abundance at the sediment from fish ponds (Lastauskienė et al. 2021). However, these orders were never detected in mesophilic aquatic habitats before.

Additionally, other biological phenomena may occur due to the mixing of water layers and nutrients in lakes, such as algal or protists blooms (Sharma et al. 2019). Indeed, the fourth most dominant phylum in the water samples from that system was Cyanobacteria (Figure 4). Other groups highly abundant and previously reported from freshwater lakes or groundwater were Chloroflexi (Kadnikov et al. 2020; Mehrshad et al. 2018), Acidobacteria (Zimmermann et al. 2012) and Ignavibacteriae (Iino 2014). The abundance of these groups tended to decrease at the outflow compared to the inflow water (*p* > 0.05), probably due to the physicochemical changes that water exhibits inside the fish rearing system. Contrarily, the heterotrophic bacteria belonging to Candidatus Sacchaibacteria (Xiujie et al. 2019), Actinobacteria (Boubekri et al. 2022) and algae (Brookfield et al. 2021) seem to benefit from the conditions inside the aquaculture system and increase in abundance at the water column (Figure 9). Major microbial community shifts happen when the availability of organic matter and oxygen create a beneficial environment for the growth of heterotrophic bacteria. The heterotrophs would then outcompete the nitrifiers and the nitrification and/or denitrification rates would decrease (Michaud et al. 2006). Thus, the abundance of groups such as Nitrospirae (Mehrani et al. 2020), Woesearchaeota (Liu, Wang, and Gu 2021) or Planctomycetes (Storesund et al. 2020), which rely on anoxic conditions to metabolise these nutrients, decreased at the outflow water (*p* > 0.05) (Figure 9). However, certain bacterial species are able to live as free‐living cell and/or attach to a substrate depending of the environmental conditions (Li et al. 2019; Schoina et al. 2022). This change of niche in aquaculture systems depends on several processes that cause major changes on water parameters such as the feeding regime. The free‐living bacteria would benefit from the suspended particles of organic matter while the cells attached forming part of the biofilm would be better protected against external factors (Balcázar, Subirats, and Borrego 2015). The raising interest to study the mechanisms behind biofilm formation and the role of such structures in aquaculture is due to its implications in water quality (Pandey, Bharti, and Kumar 2014). Some microorganisms colonising the surfaces and sediment of the tanks are able to aerobically and/or anaerobically remove the products that typically accumulate at the aquaculture system, which are mainly ammonium, nitrite, nitrate, phosphate and organic matter (Thompson, Abreu, and Wasielesky 2002). For example, the abundance of Chloroflexi, Nitrospirae, Proteobacteria, Acidobacteria, Firmicutes or Verrucomicrobia tended to decrease at the outflow water in our study (Figure 9), and are the most abundant microorganisms encountered in the tank sediment of other freshwater aquaculture systems (Bruno et al. 2023; Lastauskienė et al. 2021; Yu et al. 2021).

### 4.3 Distribution of Potentially Pathogenic Bacteria

Environmental factors that define water quality can influence the water microbiome, which interacts with the fish gut microbiome (Bruno et al. 2023). The abundance of potentially pathogenic bacteria could offer further insights into microbiome‐host interactions and the recurrence of fish infections in an aquaculture system. Results regarding pathogenic bacterial species inside two aquaculture systems revealed accurately the distribution and abundance of those agents to genus level. The sequenced 16 s rRNA region showed reliable results for the analyses of phyla, family and genera. Additionally, it was possible to detect and identify microorganisms which are very challenging to cultivate using classical microbiology, such as the intracellular pathogens *Rickettsia* sp. (Tello‐Martin et al. 2018; Yuksel, Thompson, and Adams 2006) and *Chlamydia* sp. (Stride, Polkinghorne, and Nowak 2014), whose abundance was significantly higher at Frauenmark (Table 4, Figure 8). In both farms, *Flavobacterium* sp. was the most abundant genus in the inflow and outflow water, followed by *Pseudomonas* sp. (Table 4, Figure 10). *Flavobacterium* sp. infections in salmonids and other fish species has been widely reported (Decostere, Haesebrouck, and Devriese 1998; Einarsdottir et al. 2020; Moore et al. 2014; Van Vliet, Loch, and Faisal 2015; Zhao et al. 2020), as well as *Pseudomonas* sp. infections (Altinok, Kayis, and Capkin 2006). The high abundance of such genera could be due to the high amount of nutrients in the water, the capability of the bacteria to form biofilm, or the overestimation of ASVs counts. Members of *Flavobacterium* sp. and *Pseudomonas* sp. have a versatile metabolism that enables them to use several carbon sources to obtain the energy (Arai 2011; Gavriilidou et al. 2020; Kampers et al. 2021). Thus, they benefit of the high amount of organic matter present inside the fish farms. *Flavobacterium* sp. can also form biofilm, which assures the bacterium's survival under adverse conditions and potentially becomes a stable component in calm waters such as ponds (Ríos‐Castillo et al. 2018). Such biofilm might also be easily formed on materials that are commonly used for farming activities, such as stainless steel, plastic, glass, or wood (Ríos‐Castillo et al. 2018), and are regularly used in different ponds on the same farm. To avoid cross‐contamination and biofilm formation of bacteria between different ponds is important to prevent the reoccurrence of disease in fish. Additionally, a change in the fish feeding regime could also reduce the amount of organic matter in the system, and diminish the abundance of the detected heterotrophic pathogens. Indeed, fish farms act as reservoirs of pathogens by ensuring that certain bacterial strains persist over seasons at the biofilm or at the hosts (Cai and Arias, 2017; Shamsi et al. 2021). The monitoring of such aquaculture farms could favour the effective treatment to control such pathogens, and decrease the spreading of pathogens to the wild populations.

Despite some limitations that are being discussed, the sequencing for the 16 s rRNA gene fragment is a rapid low‐cost method (Váradi et al. 2017). In this study, we present a method that is reproducible and describes the microbiome dynamics of two fish farms and includes an overview of potential pathogens present in the system. Molecular techniques are currently reasonably priced and more advanced by offering the possibility to sequence the whole 16 s rRNA gene and obtaining a taxonomic identification until species level. Thus, the reproducibility of this study could serve as a tool to support management strategies in aquaculture systems, within the aim of improving the water quality. Similarly, the description of potential pathogens could be used to find effective treatments against early detected disease infections.

## Author Contributions

**Júlia Clols‐Fuentes:** visualization, writing – original draft, writing – review and editing, formal analysis, investigation, resources. **Julien A. Nguinkal:** methodology, software, data curation, writing – review and editing. **Patrick Unger:** conceptualization, funding acquisition, project administration, writing – review and editing. **Bernd Kreikemeyer:** validation, supervision, resources, writing – review and editing. **Harry W. Palm:** conceptualization, writing – review and editing.

## Conflicts of Interest

The authors declare no conflicts of interest.

## Supporting information


**Figure S1:** Boxplot of Shannon‐Wiener Diversity (A) and Chao1 Alpha‐diversity (B) from the water microbiome in Frauenmark and Neubrandenburg. The boxes denote interquartile ranges (IGR) between the first and third quartiles, and the horizontal line inside the box defines the median. Whiskers represent the lowest and highest values within 1.5‐fold IGR from the first and third quartiles.

**Figure S2:** PCoA (Principal Coordinates Analysis) based on Bray‐Curtis distances between samples from the two different locations. Two distinct groups are observed; one group represents the samples from Frauenmark (red dots), and the other represents samples from Neubrandenburg (blue dots).

## Data Availability

The data that support the findings of this study are openly available in the National Center for Biotechnology Information (NCBI) at https://www.ncbi.nlm.nih.gov/bioproject/PRJNA1044397.
