# Supplementary figures and images for: Bacterial Communities From Two Freshwater Aquaculture Systems in Northern Germany
Source: Environ Microbiol Rep. 2024 Dec 15;16(6):e70062. doi: 10.1111/1758-2229.70062 (PMC11646623; doi:10.1111/1758-2229.70062)

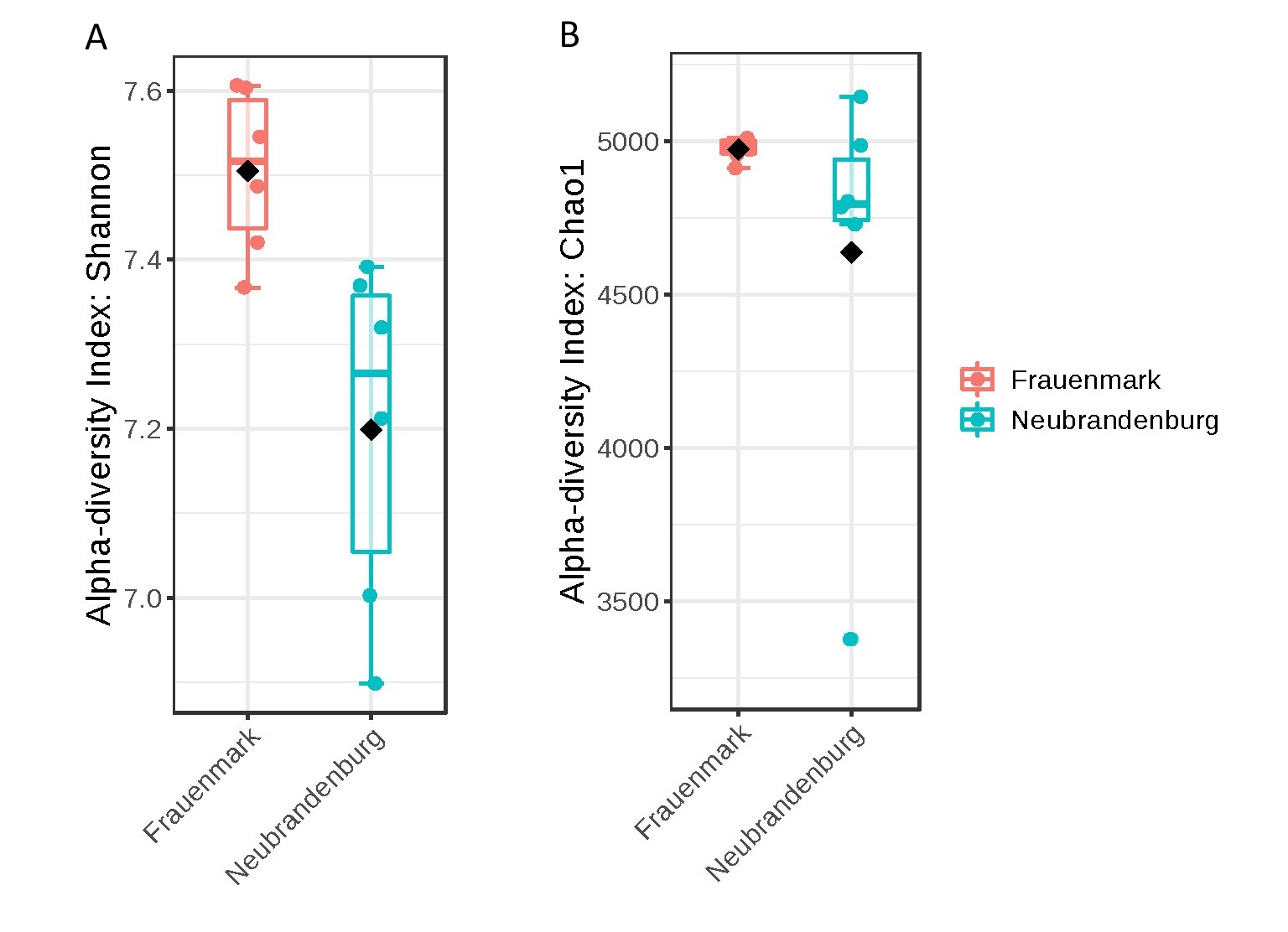

Supplement: Supplementary file 1 — Figure S1: Boxplot of Shannon‐Wiener Diversity (A) and Chao1 Alpha‐diversity (B) from the water microbiome in Frauenmark and Neubrandenburg. The boxes denote interquartile ranges (IGR) between the first and third quartiles, and the horizontal line inside the box defines the median. Whiskers represent the lowest and highest values within 1.5‐fold IGR from the first and third quartiles. [file EMI4-16-e70062-s002.jpg]

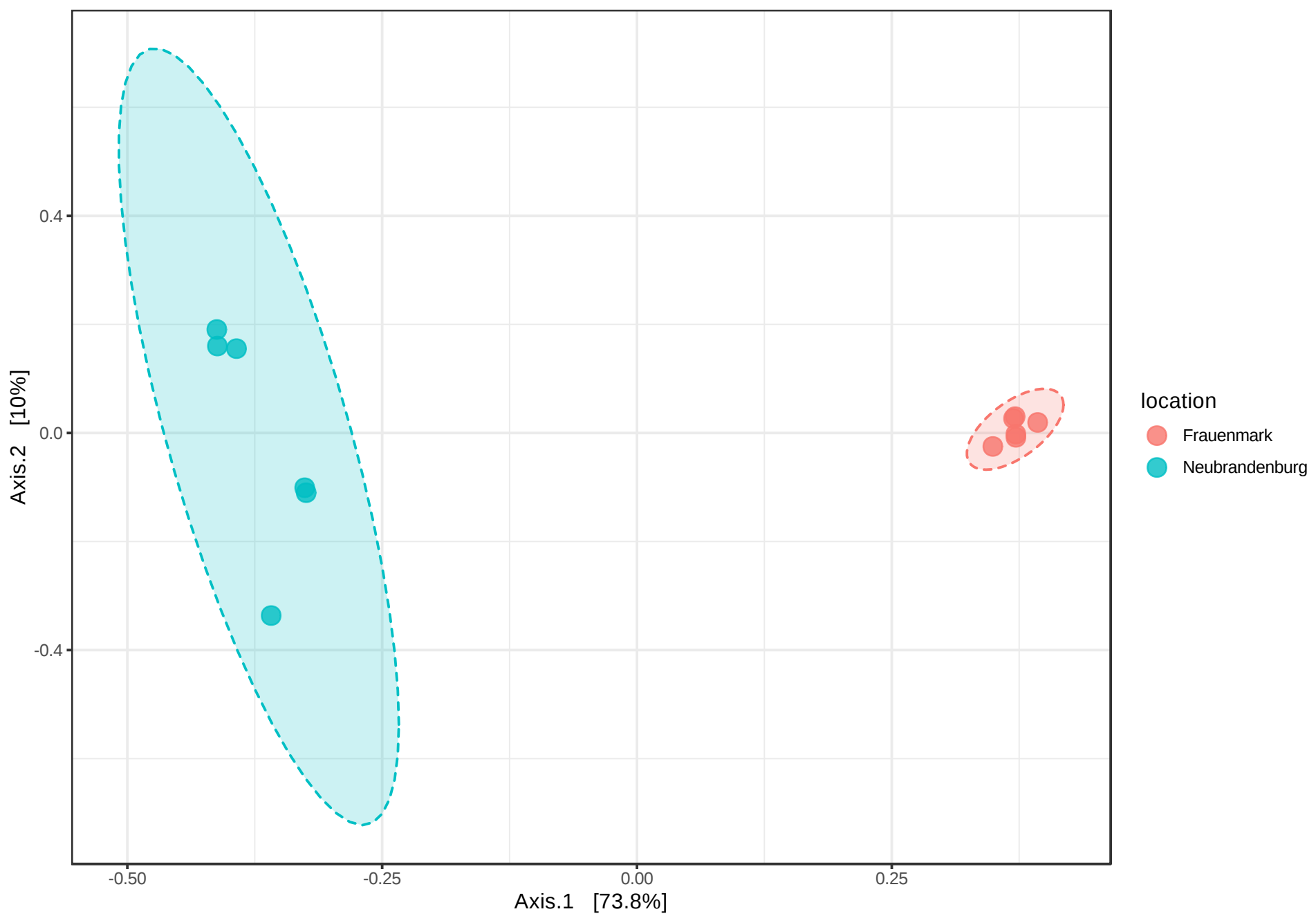

Supplement: Supplementary file 2 — Figure S2: PCoA (Principal Coordinates Analysis) based on Bray‐Curtis distances between samples from the two different locations. Two distinct groups are observed; one group represents the samples from Frauenmark (red dots), and the other represents samples from Neubrandenburg (blue dots). [file EMI4-16-e70062-s001.pdf]
